# New Boron Analogues of Pyrophosphates and Deoxynucleoside Boranophosphates

**DOI:** 10.1155/MBD.2001.145

**Published:** 2001

**Authors:** Kamesh Vyakaranam, Geeta Rana, Narayan S. Hosmane, Bernard F. Spielvogel

**Affiliations:** 1 Department of Chemistry and Biochemistry Northern Illinois University DeKalb IL 60115-2862 USA; 2 Metallo-Biotech International, Inc. 663 Teal Court DeKalb IL 60115-6201 USA

## Abstract

Tetraethyldicyanoborane pyrophosphate (**2**) and 3'-(diethylphosphite-cyanoborano)-5'-dimethoxytrityl.N^4^-benzoyl-deoxycytidine (**3**) have been synthesized in 70% and 76% yields, respectively. The compatibility of the substituted boranophosphates with common protecting groups is hereby demonstrated.

Boron containing biologically active compounds, such as nucleosides and nucleotides ^1-6^ and amino acids ^7-9^ are important due to their potential therapeutic activity, research and diagnostic applications. Many boron containing compounds have shown promising activity as anticancer, ^10.^ ^11.^ ^12^ antiinflammatory,^13^ and  antiosteoporotic ^13^agents. Oligonucleotdes in which a non-bridging oxygen atom is replaced by a borane(BH_3_) group are a very important class of modified nucleic acids. ^1.^ ^3.^ ^14-16^ The BH_3_ group is isoelectronic with oxygen in natural oligonucleotides and isoelectronic and isostructural with the oligonucleotide methyl phosphonates, which are nuclease resistant. On the other hand, the α-borano triphosphates are good substrates for DNA polymerases and incorporation of boranophosphates into DNA causes an increase in the resistance to exo- and endonucleases ^2.^ ^17a^ as compared to non-modified DNA. There are also notable applications of the α-borano triphosphates in PCR sequencing ^17a^ and nucleic acid detection ^17b^.


### NEW BORON ANALOGUES OF PYROPHOSPHATES AND DEOXYNUCLEOSIDE BORANOPHOSPHATES

Kamesh Vyakaranam[1], Geeta Rana[1], Narayan S. Hosmane*[1], and Bernard F. Spielvogel*[1,2]

[1] Department of Chemistry and Biochemistry, Northern Illinois University, DeKalb, IL 60115-2862, USA.
[2] Metallo-Biotech International, Inc., 663 Teal Court, DeKalb, IL 60115-6201, USA

### ABSTRACT

Tetraethyldicyanoborane pyrophosphate (**2**) and 3'-(diethylphosphite-cyanoborano)-5'-dimethoxytrityl-N$^4$-benzoyl-deoxycytidine (**3**) have been synthesized in 70% and 76% yields, respectively. The compatibility of the substituted boranophosphates with common protecting groups is hereby demonstrated.

Boron containing biologically active compounds, such as nucleosides and nucleotides [1-6] and amino acids [7-9] are important due to their potential therapeutic activity, research and diagnostic applications. Many boron containing compounds have shown promising activity as anticancer,[10, 11, 12] antiinflammatory,[13] and antiosteoporotic [13]agents. Oligonucleotides in which a non-bridging oxygen atom is replaced by a borane ($BH_3$) group are a very important class of modified nucleic acids. [1, 3, 14-16] The $BH_3$ group is isoelectronic with oxygen in natural oligonucleotides and isoelectronic and isostructural with the oligonucleotide methyl phosphonates, which are nuclease resistant. On the other hand, the α-borano triphosphates are good substrates for DNA polymerases and incorporation of boranophosphates into DNA causes an increase in the resistance to exo- and endonucleases [2, 17a] as compared to non-modified DNA. There are also notable applications of the α-borano triphosphates in PCR sequencing [17a] and nucleic acid detection.[17b]

### Figure 1. Isoelectronic Nucleic Acid Backbones

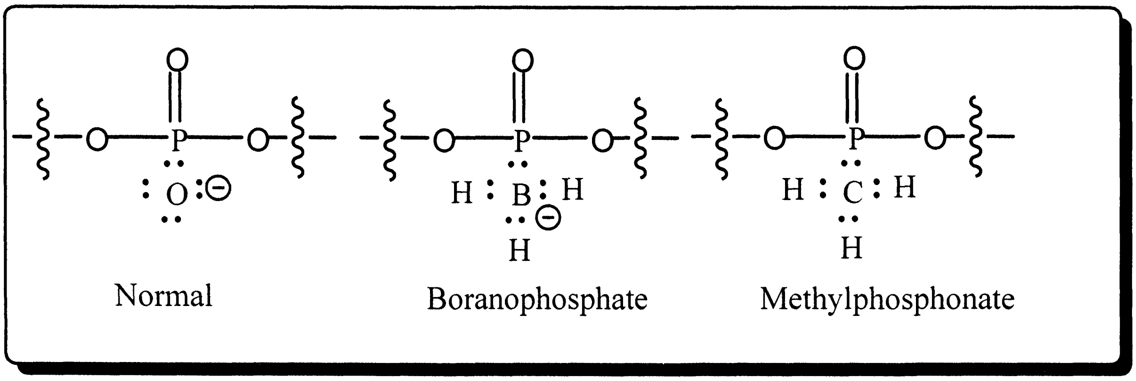

Although considerable effort and progress has been made with the boranophosphate linkage, numerous limitations with this moiety still exist, especially in chemical syntheses. Thus, the highly reducing borane group causes base degradation and incompatibility with commonly used protecting groups in modified oligonucleotide synthesis.[3(a), 18a] Likewise, the $BH_3$ moiety has severe toxicity implications. [18b] We have been intensively investigating the attachment of substituted boranes such as $BH_2X$, where X = COOR, C(O)NR'H, CN, etc., to phosphorus in biologically important molecules. Here we report the use of substituted boranes in the syntheses of boronated pyrophosphates and nucleic acids which promises to greatly expand the scope of this class of nucleic acids.

Reaction of the potassium salt[20] of diethylphosphite-cyanoborane **1** with methanesulfonyl chloride gave tetraethyldicyanoborane pyrophosphate **2** as shown in **Scheme 1**. Purification was achieved by column chromatography using ethyl acetate as an eluent. The yields range from 65-71% [21]. Analysis by $^{31}P$ NMR spectroscopy showed a quartet of triplet at δ 80.21 ppm corresponding to the cyanoborane phosphate group. The $^{11}B$ NMR spectrum of **2** contained a doublet of triplet at δ – 40.58 ppm corresponding to cyanoborane phosphate group. IR spectrum of the purified product showed a broad band at 2409 $cm^{-1}$ corresponding to B-H stretch and a sharp peak at 2361 $cm^{-1}$ consistent with CN stretch.

**REACTION SCHEME 1:**

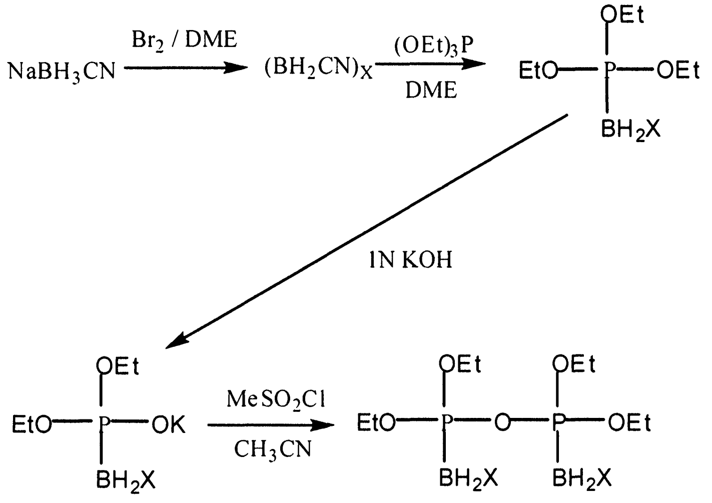

1 X =CN 2 X =CN

**REACTION SCHEME 2:**

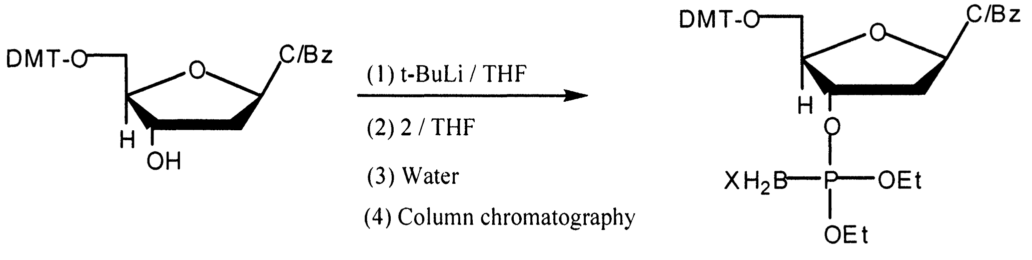

3 X =CN

The preparation of $K^-$ $(RO)_2PBH_3^-$ salt and analogous tetramethyl-boranopyrophosphate was previously reported by Imamato *et. al.* [22], and demonstrated to have good versatility in the preparation of boranophosphate compounds. However, these species may still be of limited use because of the presence of highly reducing $BH_3$ moiety. Convincing evidence that *substituted* boranes with less reducing power can mitigate many of the problems caused by the $BH_3$ group is demonstrated by the synthesis of **3**. In this instance, the 5'-trityl protected group is retained in the synthesis whereas with borane unit, $BH_3$, it is completely removed. [3a,18] Thus, the reaction of 5'-protected 2'-deoxycytidine, Benzoyl (Bz), dimethoxytrityl (DMT) deoxycytidine, with pyrophosphate **2** (**Scheme 2**), followed by evaporation of the solvent resulted in the formation of 3'-(diethylphosphite-cyanoborano)- 5'-dimethoxytrityl-$N^4$-benzoyl-deoxycytidine **3**. This crude product was purified by flash chromatography and obtained in 75% yield (by $^1H$ NMR) [23]. Analysis by $^{31}P$ NMR spectroscopy showed a quartet of triplet at δ 68.12 ppm corresponding to the cyanoborane phosphate group. The $^{11}B$ NMR spectrum of **2** contained a doublet of triplet at δ – 39.63 ppm corresponding to cyanoborane phosphate group. IR spectrum of the purified product showed two broad bands at 2762 and 2701 $cm^{-1}$, corresponding to B-H stretch, and a sharp peak at 2295 $cm^{-1}$ is consistent with CN stretch. The DMT protecting group and boranophosphate were incompatible in previous synthetic procedures.[3a,18] However, with the use of the less reducing cyanoborane, the DMT nucleoside was isolated in 75% yield using the procedure described here. Substituted boranes are less reducing than $BH_3$ moiety and should also inhibit the base degradation. In fact, cyanoborane adducts of various N positions of purine and pyrimidine nucleosides have been prepared with the bases not reduced or degraded. [3(b)]

Thus, in view of the great importance of phosphate moieties (pyrophosphates, nucleic acids, etc.) in biological processes, the availability of boronated phosphates, such as **2** and **3**, is expected to be of considerable interest. Work on a variety of such species is currently underway in our laboratories.

**ACKNOWLEDGMENT.** This work was supported by grants from the National Science Foundation (CHE-9988045), the donors of the Petroleum Research Fund, administered by the American Chemical Society, and Northern Illinois University through Presidential Research Professorship (to NSH).

### REFERENCES

* Corresponding author, Fax (815)753-4802, email: nhosmane@niu.edu

1. Hasan, A.; Li, H.; Tomasz, J.; Shaw, B.R.; *Nucl. Acid Res.* **1996**, *24*, 2150-2157.
2. (a) Li, H.; Porter, K.; Huang, F.; Shaw, B.R.; *Nucl. Acid Res.* **1995**, *23*, 4495-4501. (b) Sood, A.; Shaw, B.R.; Spielvogel, B.F.; *J. Am. Chem. Soc.* **1990**, *112*, 9000-9001. (c) Sood, A.; Spielvogel, B.F.; Shaw, B.R.; *J. Am. Chem. Soc.* **1989**, *111*, 9234-9235.
3. Spielvogel, B.F.; Sood, A.; Shaw, B.R.; Hall, I.H.; Fairchild, R. G.; Laster, B.H.; Gordon, C.; *Progress in Neutron Capture therapy for Cancer* **1992**, 211-213.
4. Shaw; B.R.; Madison, J.; Sood, A.; Spielvogel, B.F.; *Methods in Mol. Biol.* **1993**, *20*, 225-243.
5. Kane, R.R.; Drechsel, K.; Hawthorne, M.F.; *J. Am. Chem. Soc.* **1993**, *115*, 8853-8854.
6. Spielvogel, B.F.; Sood, A.; Shaw, B.R.; Hall, I.H.; *Pure Appl. Chem.* **1991**, 63, 415-418.
7. Spielvogel, B.F.; Sood, A.; Tomasz, J.; Shaw, B.R.; Karthikeyan, S.; *Neutron Capture Ther.* **1993**, 361-365.
8. Kane, R. R.; Pack, R. H.; Hawthorne, M.F.; *J. Org. Chem.* **1993**, *58*, 991-992.
9. Hall, I.H.; Hall, E. S.; Chi, L.; Shaw,B. R.; Sood, A.; Spielvogel, B. F.; *Anticancer Res.* **1992**, *12*, 1091-1098;
10. Sood, A.; Spielvogel, B.F.; Shaw B.R.; Carlton, L.D.; Burnham, B.S.; Hall, E.S. Hall, I.H.; *ibid.* **1992**, *12*, 335-334.
11. Sood, A.; Shaw, B.R.; Spielvogel, B.F.; Hall, E.S.; Chi, L.K.; and hall, I.H.; *Pharmazie* **1992**, *47*, 833-838.
12. Spielvogel, B.F.; Sood, A.; Shaw, B.R.; Hall, I.H.; *Curr. Top. Chem. Boron Proc. Int. Meet. Boron Chem.*, 8$^{th}$, **1994**, pp. 193-198.
13. Rajendran, K.G.; Burnham, B.S.; Chen, S.Y.; Sood, A.; Spielvogel, B. F.; Shaw, B.R.; Hall, I.H. *J. Pharm. Sci.* **1994**, 83, 1391-1395.
14. Tomasz, J.; Shaw, B.R.; Porter, K.W.; Spielvogel, B.F.; Sood, A. *Angew. Chem., Int. Ed. Engl.* **1992,** 31, 1373-1375.
15. Krzyzanowska, B. K.; He, K.; Hasan, A.; Shaw, B.R. *Tetrahedron* **1998**, *54*, 5119-5128.
16. He, K.; Hasan, A.; Krzyanowska, B. K.; Shaw, B.R. *J. Org. Chem.* **1998**, *63*, 5769-5773.
17. (a) Porter, K. W.; Briley, J.D.; Shaw, B.R.; *Nucleic Acid Res.* **1997**, *25*, 1611-1617. (b) Spielvogel, B. F.; Powell, W.; Sood, A. *Main Group Metal Chemistry*, **1996**, *19*, 699-704.
18. (a) Higson, A. P.; Sierzchala, A.; Brummel, H.; Zhao, Z.; Caruthers, M. H. *Tetrahedron Letters*, **1998**, *39*, 3899-3902. (b) Levinskas, G. J., Boron, Metalloboron compounds and Boranes, Chap. 8, *Interscience*, **1964**.
19. (a) Eriksson, B.; Oberg, B.; Wahren, B. *Biochim. Biophys. Acta* **1982**, *696*, 115-123. (b) Eriksson, B.; Larsson, A.; Helgstrand, E.; Johansson, N. G.; Oberg, B. *Biochim. Biophys. Acta* **1980**, *607*, 53-64. (c) Eriksson, B.; Tao, P. Z.; Wahren, B.; Oberg, B. Proc. *Int. Confr. Chemother.*; **13th, 1983**, *6*, 114/29-114/32.
20. Sood, A.; Sood, C.K.; Hall, I.H.; Spielvogel, B.F. *Tetrahedron*, **1991**, *47*, 6915-6930.
21. Methanesulfonyl chloride (0.96g, 8.34mmol) was added to a solution of the potassium salt of diethylphosphite-cyanoborane (3.0g, 13.9mmol) in acetonitrile (50 mL) at $0^0C$ and the mixture was stirred at room temperature for 4 h. The precipitated solid was removed by filtration, and the filtrate was concentrated under reduced pressure. The residual oil was passed through a short column of silica gel with ethyl acetate to give 3.25g (70%) compound 2 as a colorless oil. $^1H$ NMR (200 MHz; DMSO reference to TMS) δ 1.20 (sh, t, 3H), 3.85 (sh, m, 2H); $^{11}B$ NMR (64.21 MHz; DMSO reference to $BF_3.OEt_2$) δ -40.58 (d of t, $^1J_{BP}$ = 160.4 Hz); $^{13}C$ NMR (50.32 MHz; DMSO reference to TMS) δ 16.9 (d of d, $^3J_{PC}$ = 4.0 Hz, $CH_3$; 57.71, d of d, $^2J_{PC}$ = 4.9Hz, $CH_2$). $^{31}P$ NMR (81.01 MHz;

DMSO reference to $H_3PO_4$) δ 80.21 (q of t, $J_{PB}$ = 163 Hz); IR (KBr pellet) 2409, 2361 ν(BH); 2209 ν(CN). Anal. Calcd for $C_{10}H_{24}P_2O_5B_2N_2$: C, 35.74; H, 7.2; N, 8.33. Found: C, 35.01; H, 7.9; N, 8.56. This compound decomposes at 120 °C.

22. Imamoto, T.; I.; Nagato, E.; Wada, Y.; Masuda, H.; Yamaguchi, K.; Uchimaru, T. *J. Am. Chem. Soc.* **1997**, *119*, 9925-9926.
23. Benzoyl-Dimethoxytrityl-deoxycytidine (250 mg, 0.4mmol) was dissolved in 15 mL of THF and the solution was cooled to −78 °C. After 30 minutes, t-BuLi (1.0mL, 1.25mmol) was added to the reaction mixture and the reaction was carried out for 30 minutes. Tetraethyldicyanoborane pyrophosphate (200mg, 0.5mmol) dissolved in 10mL of THF was added to the solution and reaction continued for 18 h. The solvent was evaporated under reduced pressure. To the crude extract, 25 mL of water was added and solution was stirred for 5 h. The solid was filtered off and purified by chromatography using ethyl acetate/ hexane (9:1) to give 0.24g (75.7%) of compound 3. $^1$H NMR (200 MHz; DMSO reference to TMS) δ 8.23 (m, 5H, Ar-H), 7.69(br, 13H, Ar-H), 7.65 (d, 2H, H-5, H-6), 6.18 (t, 1H, H-1'), 2.21, 2.15 (m, 2H, H-2'), 4.0 (m, 1H, H-3'), 5.37 (m, 1H, H-4'), 3.67, 4.05 (m, 2H, H-5'), 3.95 (q, 4H), 1.3 (t, 6H); $^{11}$B NMR (64.21 MHz, DMSO reference to $BF_3.OEt_2$) δ -39.63 (d of t, $^1J_{BP}$ = 178.6 Hz ); $^{31}$P NMR (81.10 MHz; DMSO reference to $H_3PO_4$) δ 68.12 (q of t, $J_{PB}$ = 163Hz). IR (KBr pellet) 2762, 2701 ν(BH); 2295 ν(CN). Anal. Calcd for $C_{42}H_{46}O_9N_4BP$: C, 63.63; H, 5.80; N, 7.07. Found: C, 62.89; H, 6.10; N, 6.42.

**Received: May 28, 2001 – Accepted: June 20, 2001 – Accepted in publishable format: June 20, 2001**

